# Molecular characterization of genotype II African swine fever virus circulating in three provinces of Indonesia, 2021–2023

**DOI:** 10.14202/vetworld.2026.1010-1026

**Published:** 2026-03-15

**Authors:** Atik Ratnawati, Risza Hartawan, Indrawati Sendow, Sumarningsih Sumarningsih, Muharam Saepulloh, Simson Tarigan, Harimurti Nuradji, Ni Luh Putu Indi Dharmayanti, I Wayan Teguh Wibawan, Ni Luh Putu Ika Mayasari

**Affiliations:** 1 Doctoral Student of Veterinary Biomedical Sciences, Graduate School, IPB University, Bogor, 16680, Indonesia; 2 Research Center for Veterinary Science, Research Organization for Health, National Research and Innovation Agency, Cibinong, 16911, Indonesia; 3 Division of Medical Microbiology, School of Veterinary Medicine and Biomedical Sciences, IPB University, Bogor, 16680, Indonesia

**Keywords:** African swine fever, ASFV genotype II, ASFV Indonesia, *E183L* gene, genetic characterization, I73R I329L intergenic region, molecular epidemiology, *p72* gene

## Abstract

**Background and Aim:**

African swine fever (ASF) is a highly contagious and often fatal hemorrhagic viral disease affecting domestic and wild pigs. Since its emergence in Indonesia in 2019, ASF virus (ASFV) has spread across multiple islands and become endemic in numerous provinces. Previous molecular investigations have consistently identified genotype II as the predominant circulating genotype; however, these studies offered limited information on intra-genotypic diversity, geographic distribution, and temporal genetic stability across distant regions. The present study aimed to determine the genotype and evaluate genetic variations of representative ASFV strains responsible for outbreaks in East Kalimantan, North Sumatra, and East Nusa Tenggara provinces during 2021–2023. Molecular characterization was performed using the partial *B646L* (*p72*) gene, the complete *E183L* (*p54*) gene, and the tandem repeat sequences within the intergenic region between *I73R* and *I329L* genes. To the best of our knowledge, this represents the first multi-province and multi-year analysis of ASFV in Indonesia employing this combined molecular approach.

**Materials and Methods:**

A total of 33 clinical specimens (organs and swabs) collected from affected backyard pigs and farm environments in Berau City (East Kalimantan), Pematang Siantar (North Sumatra) and Kupang (East Nusa Tenggara) were screened for ASFV by quantitative polymerase chain reaction (PCR) targeting the *B646L* (*p72*) gene. Five representative samples, selected based on cycle threshold values and geographic representation, were subjected to conventional PCR amplification and Sanger sequencing of the three target genomic regions. Nucleotide sequences were assembled, aligned, and analyzed phylogenetically using neighbor-joining and minimum evolution methods with 1 000 bootstrap replicates. Amino acid translations and tandem repeat motif comparisons were conducted to detect insertions, deletions, or sequence polymorphisms relative to reference strains.

**Results:**

ASFV DNA was detected in all 33 specimens. Phylogenetic analyses of partial *B646L* (*p72*) and full-length *E183L* (*p54*) sequences unambiguously classified the five Indonesian isolates within genotype II, clustering closely with contemporary Asian genotype II strains. Sequence analysis of the *E183L* (*p54*) gene revealed a previously unreported insertion of five amino acids (PVTDN motif) at positions 121–125 in the two isolates originating from North Sumatra; this duplication was absent from all other Indonesian and Southeast Asian genotype II sequences examined. Intergenic region analysis demonstrated that all isolates belonged to the IGR II variant, characterized by two identical 10-nucleotide GGAATATATA tandem repeat units. This motif showed 100% identity across the 2021–2023 samples and matched the configuration reported from the initial 2019 outbreak in North Sumatra, indicating sustained genetic homogeneity.

**Conclusion:**

The ASFV strains responsible for outbreaks in three geographically distant Indonesian provinces from 2021 to 2023 belonged exclusively to genotype II and exhibited low genetic diversity. The novel PVTDN insertion identified in the *E183L* (*p54*) gene of North Sumatra isolates constitutes a unique molecular marker with potential utility for lineage tracing. The identical IGR II profile across provinces and years confirms spatial and temporal genetic stability of the circulating virus. These findings underscore the value of integrated analyses of *B646L* (*p72*), *E183L* (*p54*), and the *I73R*/*I329L* intergenic region for refining the molecular epidemiology of ASFV and provide critical baseline data to support enhanced surveillance, diagnostic development, and control strategies in Indonesia.

## INTRODUCTION

African swine fever (ASF) is a deadly, contagious, and hemorrhagic disease that affects both domestic and wild pigs [1]. ASF virus (ASFV) is highly virulent and remains a global threat because no effective vaccines are available to prevent the disease [2]. Since the 1920s, ASF has caused huge economic losses to the swine industry worldwide and is a notifiable disease to the World Organization for Animal Health [3]. ASFV is a large DNA virus belonging to the family Asfarviridae [4]. ASFV is transmitted through the sylvatic and domestic cycles or between sylvatic and domestic pigs [5]. ASFV spreads between wild pigs in the sylvatic cycle. Warthogs serve as reservoir hosts for ASFV and maintain persistent infections without exhibiting overt clinical symptoms. Soft ticks of the genus Ornithodoros function as ASFV vectors, facilitating viral persistence within the sylvatic cycle and transmission to domestic pigs [5, 6]. The domestic cycle involves direct transmission between domestic pigs or through contaminated feed without the involvement of sylvatic hosts [7]. Transmission of ASFV from the sylvatic cycle to domestic pigs occurs through tick bites, consumption of contaminated warthog carcasses by domestic pigs, or contact with warthog feces [8, 9]. Once ASFV is introduced to domestic pigs, the virus propagates through direct contact between infected and susceptible individuals, feeding of contaminated meat, or via fomites such as contaminated clothing, footwear, equipment, and vehicles [5].

Currently, ASFV strains from different countries are classified into 24 genotypes based on partial *B646L* (*p72*) gene sequences [10, 11]. All 24 ASFV genotypes are present in Africa; only genotypes I and II have been reported in European and Asian countries [12, 13]. ASFV genotype I was introduced into Europe in 1957 and reintroduced from Portugal after re-entry in 1960 [14]. In Europe, ASFV genotype II was first detected in Georgia in 2007 [15, 16]. In 2014, it spread to neighboring countries, including Russia, and EU member countries in 2014. In September 2018, Belgium reported ASF in two wild boars (Sus scrofa) caused by genotype II isolates [17–19]. In Asia, ASF was first reported in China in August 2018 [20, 21]. From China, ASFV rapidly spread to neighboring countries, including Mongolia, Vietnam, Cambodia, Laos, the Philippines, Timor-Leste, South and North Korea, and Indonesia, in 2019 and to Myanmar in 2021 [13, 18, 22, 23].

The ASFV genome is a complex DNA genome ranging in length from 170 to 192 kbp, depending on the virus strain [23–25]. Sequence analysis of distinct ASFV genomic regions has proven very useful for identifying the origin and transmission pathways of ASF during outbreaks [26]. Based on the ASFV p72 major capsid protein gene *B646L*, 24 distinct ASFV genotypes (I–XXIV) have been described. Analysis of additional genes provides higher resolution for distinguishing between closely related isolates [10, 11]. The p54-encoding gene *E183L* increased the intragenotypic resolution of standard *B646L* (*p72*) genotyping. On the largest branch of *B646L* (*p72*) genotype I, the *E183L* (*p54*) genotype can be further divided into four discrete subclusters: Ia, Ib, Ic, and Id [27]. The central variable region (CVR) within the *B602L* gene provides additional information on relationships among isolates at the genotype, country, and regional levels [28–30]. Recent studies have demonstrated the value of tetrameric/tandem repeat sequences (TRS) located in the intergenic region between the *I73R* and *I329L* (*I73R*/*I329L*) genes in determining the origin and mapping the spread of closely related ASFV isolates [20, 31]. The *B646L* (*p72*) and *E183L* (*p54*) genes are essential for genotype classification, whereas the CVR of *B602L* and IGR between *I73R*/*I329L* genes provide insights into genetic diversity and strain variations, which are crucial for understanding the diversity and epidemiology of ASFV in different regions [13, 32]. While the focus on *B646L* (*p72*), *E183L* (*p54*), IGR between *I73R*/*I329L*, and *B602L* (CVR) provides valuable insights into ASFV diversity, other genetic factors and environmental influences that may also play a role in ASF epidemiology should be considered. This broader perspective can enhance our understanding of ASFV dynamics and inform more effective control strategies [13, 33].

The first ASF outbreak was reported in 2019 at a backyard pig farm in North Sumatra Province, Indonesia, in 2019 [22]. Since then, serial ASF outbreaks in domestic pigs have been continuously reported and have become endemic across the country. According to the FAO report, ASF has been reported in 32 out of 34 provinces in Indonesia (Sumatra, Java, Bali, Sulawesi, Kalimantan, and Nusa Tenggara). Several studies have been conducted to determine the ASFV genotypes circulating in Indonesia. All of these studies indicated that ASFV-causing outbreaks belonged to genotype II, which is the most predominant genotype in Indonesia [34, 35]. However, these studies provide limited information on the virus’s molecular properties and epidemiology [36].

In Indonesia, molecular data on ASFV following the initial 2019 incursions remain limited, and most reports describe single outbreaks or short temporal windows. To date, no multi-year and molecular characterization of ASFV circulating across different provinces has been published. The present study addresses this gap by analyzing ASFV-positive samples collected from outbreaks in East Kalimantan, North Sumatra, and East Nusa Tenggara between 2021 and 2023. This represents the first multi-year (2021–2023) dataset covering multiple island regions of Indonesia. Furthermore, this study documents for the first time a unique five amino acid insertion (PVTDN) in the *E183L* (*p54*) gene of two Indonesian genotype II isolates. This insertion has not been reported in previously available Indonesian sequences from 2019 to 2023 or in other Southeast Asian genotype II strains. It resembles insertions found in African genotypes (XV, XVI, XIII) but occurs independently. Identification of this novel molecular feature provides an additional marker that may support future lineage tracing and enhance the understanding of ASFV microevolution in Indonesia. Therefore, further analysis is required to provide more information on the genetic characterization and variation of ASFV strains from Indonesia [37].

Limited longitudinal and spatially comparative molecular data currently exist on ASFV genotype II circulating in Indonesia beyond the initial incursion phase, hindering accurate reconstruction of transmission networks and assessment of viral adaptation in an island nation. Accordingly, this study aimed to provide the first multi-year (2021–2023) and multi-province molecular characterization of ASFV isolates obtained from backyard pig outbreaks in East Kalimantan, North Sumatra, and East Nusa Tenggara provinces. The specific objectives were to (1) confirm continued circulation of genotype II through sequencing of the partial *B646L* (*p72*) gene, (2) evaluate intragenotypic diversity and detect any amino acid insertions or substitutions by sequencing the complete E183L (*p54*) gene, and (3) determine the IGR variant (TRS configuration in the I73R/I329L intergenic region) to assess fine-scale relatedness among strains from disparate geographic origins. Through these analyses, the work sought to establish whether ASFV populations in these three provinces exhibit genetic homogeneity indicative of a single successful introduction and sustained spread, or whether region-specific microevolutionary events (including the novel five amino acid PVTDN insertion in E183L) have occurred, thereby contributing critical information for targeted surveillance, diagnostic refinement, and long-term ASF control planning in Indonesia.

This study aimed to determine the genotypes and genetic variations of representative ASFV strains causing outbreaks in Indonesia from 2021 to 2023, based on the *B646L* (*p72*) gene segment, the full-length *E183L* (*p54*) gene, and a fragment between the *I73R*/ *I329L* genes.

## MATERIALS AND METHODS

### Ethical approval

The Animal Care and Use Ethics Committee of the Indonesian National Research and Innovation Agency approved this research (reference number 100/KE.02/SK/12/2022).

### Study area and study period

The study was conducted using diagnostic specimens of organs or swabs collected randomly by provincial veterinary service from backyard pig farms in three provinces in Indonesia from July 2021 to March 2023: Berau city (East Kalimantan Province), Pematang Siantar city (North Sumatra Province), and Kupang city (East Nusa Tenggara, also known as NTT Province). These three provinces are located on three different islands: Sumatra, Kalimantan, and Timor. During 2021–2023, three representative ASF outbreaks in backyard pig farms were officially reported by the province’s veterinary service and selected for this study.

### Sampling collection

Pigs selected for ASFV samples showed clinical signs of ASF, including fever, lethargy, anorexia, cutaneous hyperemia, hemorrhages, and sudden death in acute cases, according to a report from the provincial veterinary service. Tissue samples (5 g per organ) were collected from the spleen, kidney, heart, lung, and liver. Additionally, swab samples (oral or nasal) were collected using sterile synthetic swabs and placed in VTM. The provincial veterinary service conducted sampling using appropriate field-level biocontainment precautions, including the use of personal protective equipment, such as gloves, masks, protective clothing, and boots. Sterile instruments were changed between animals and prevent cross-contamination. Samples were transported from the field to the laboratory within 1 week of collection, maintaining a cold chain throughout transport. All samples from suspected ASFV-infected pigs were transported in sealed containers on ice packs according to the International Air Transport Association international standards regulation, using a triple packaging system, to the BSL2 level of the Virology Laboratory at the Research Center for Veterinary Science, Bogor, West Java. Samples were stored at 4°C for short-term storage before processing and were subsequently used for analysis. To avoid contamination, samples from the three provinces were received in different months and processed separately according to the standard operating protocol. Samples were collected during outbreak investigations in backyard pig farms. Each diagnostic specimen represented a single organ, blood sample, or environmental swab. Multiple specimens could be collected from the same pig (e.g., different organs), which are identified by the same laboratory code in Table 1. The environmental swab samples were included and are explicitly indicated.

**Table 1:** Detection of ASFV in organs and swabs of pigs in three Indonesian provinces during 2021–2023.

Specimen No.	Laboratory code	Year	Province/District	Sample type	Sampling unit	qPCR (Ct)
1	Smada1	2021	Berau, East Kalimantan, Indonesia	Heart	Pig1	30.59
2	Smada 2	2021	Berau, East Kalimantan, Indonesia	Kidney	Pig1	28.31
3	Smada 3	2021	Berau, East Kalimantan, Indonesia	Liver	Pig1	28.78
4	Smada 4*	2021	Berau, East Kalimantan, Indonesia	Spleen	Pig1	28.47
5	Smada 5	2021	Berau, East Kalimantan, Indonesia	Lung	Pig1	27.43
6	PSJ*	2022	Pematang Siantar, North Sumatra, Indonesia	Spleen	Pig2	23.26
7	PSJ	2022	Pematang Siantar, North Sumatra, Indonesia	Lung	Pig2	30.07
8	PSJ	2022	Pematang Siantar, North Sumatra, Indonesia	Liver	Pig2	24.65
9	PSJ	2022	Pematang Siantar, North Sumatra, Indonesia	Heart	Pig2	28.29
10	PSJ	2022	Pematang Siantar, North Sumatra, Indonesia	Kidney	Pig2	31.76
11	PSB	2022	Pematang Siantar, North Sumatra, Indonesia	Liver	Pig3	23.85
12	PSB	2022	Pematang Siantar, North Sumatra, Indonesia	Lung	Pig3	25.42
13	PSB	2022	Pematang Siantar, North Sumatra, Indonesia	Kidney	Pig3	23.38
14	PSB*	2022	Pematang Siantar, North Sumatra, Indonesia	Spleen	Pig3	23.11
15	PSB r	2022	Pematang Siantar, North Sumatra, Indonesia	Heart	Pig3	27.76
16	PSKd1	2022	Pematang Siantar, North Sumatra, Indonesia	Cage swab	Environment	25.67
17	NTT 6	2023	Kupang, NTT	Nasal swab	Pig4	22.71
18	NTT 6	2023	Kupang, NTT	Anal swab	Pig4	23.33
19	NTT 16	2023	Kupang, NTT	Anal swab	Pig5	24.62
20	NTT 17	2023	Kupang, NTT	Anal swab	Pig6	23.15
21	NTT 23	2023	Kupang, NTT	Nasal swab	Pig7	24.22
22	NTT 23	2023	Kupang, NTT	Anal swab	Pig7	23.21
23	NTT 23*	2023	Kupang, NTT	Spleen	Pig7	22.85
24	NTT 23 h	2023	Kupang, NTT	Lung	Pig7	24.26
25	NTT 23	2023	Kupang, NTT	Liver	Pig7	24.48
26	NTT 23	2023	Kupang, NTT	Kidney	Pig7	29.04
27	NTT 24	2023	Kupang, NTT	Nasal swab	Pig8	27.18
28	NTT 24	2023	Kupang, NTT	Anal swab	Pig8	29.80
29	NTT 24	2023	Kupang, NTT	Cage swab	Environment	30.64
30	NTT 32	2023	Kupang, NTT	Chopping block swab	Environment	28.99
31	NTT 32	2023	Kupang, NTT	Knife swab	Environment	28.65
32	NTT 32*	2023	Kupang, NTT	Liver	Pig9	24.78
33	NTT 32	2023	Kupang, NTT	Lung	Pig9	27.37

* Sample subjected to sequencing. Each row represents one diagnostic specimen. Laboratory code identifies the sampled pig or environmental sampling point; repeated laboratory codes indicate multiple specimen types collected from the same pig. Environmental specimens are explicitly labeled. A total of 33 specimens were obtained from 9 pigs across three outbreak locations together with four environmental swab samples. NTT = East Nusa Tenggara, qPCR = Quantitative polymerase chain reaction.

### DNA extraction

Viral DNA was extracted directly from 10% homogenized organs or swabs of diagnostic samples using the DNeasy Blood and Tissue Kit (Qiagen, Hilden, Germany) according to the manufacturer’s instructions. For nucleic acid extraction, tissue samples were processed at a 25 mg tissue per 200 µL lysis buffer ratio, and whole blood samples (200 µL) were mixed with 200 µL lysis buffer according to the extraction protocol. In brief, 10% (w/v) clarified homogenized tissue suspensions or swabs were prepared using a viral transport medium. Up to 25 mg of tissue (up to 10 mg of spleen) was cut into small pieces or swab samples, placed in a 1.5 mL microcentrifuge tube, and 180 µL of Buffer ATL (Qiagen) was added. Next, 20 µL of proteinase K was added, the mixture was vortexed, and the mixture was incubated at 55°C until the tissue was completely lysed (1–3 h). The sample was occasionally vortexed for 15 s during incubation to disperse it. Next, 200 µL of Buffer AL (Qiagen) was added to the sample, vortexed to mix, and then incubated at 70°C for 10 min. Next, 200 µL of 96% ethanol was added to the sample, and the mixture was mixed thoroughly by vortexing. The mixture from the previous step was pipetted into a DNeasy Mini Spin Column and placed in a 2 mL collection tube. Centrifuge at ≥6,000 x *g* for 1 min. The flow-through and collection tubes were discarded. The DNeasy Mini Spin Column was placed in a new 2-mL collection tube, and 500 µL of AW1 Buffer was added. The mixture was centrifuged for 1 min at ≥6000 × *g*. Discard the flow-through and collection tube. The DNeasy Mini Spin Column was placed in a 2 mL collection tube, and 500 µL of AW2 Buffer was added. The mixture was centrifuged for 3 min at 20,000 × *g* to dry the DNeasy membrane. Discard the flow-through and collection tube. The DNeasy Mini Spin Column was placed in a clean 1.5 mL microcentrifuge tube, and 100 µL of AE Buffer (Qiagen) was pipetted directly onto the DNeasy membrane. The mixture was incubated at room temperature for 1 min and then centrifuged for 1 min at ≥6000 × *g* to elute. The elution step was repeated once, as described in the previous section. A new microcentrifuge tube can be used for this purpose. The extracted nucleic acids were quantified and assessed for purity using a NanoDrop spectrophotometer based on absorbance ratios. The extracted DNA was stored at −20°C until further analysis.

### ASFV detection by quantitative polymerase chain reaction (qPCR)

**Figure 1:**
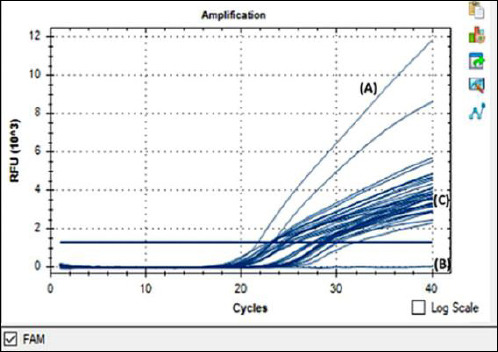
Detection of ASFV in pig organs and swab samples by real-time polymerase chain reaction. Real-time polymerase chain reaction amplification curves showing ASFV detection. The cycle threshold (Ct) value was 21.66 for the positive control (A), no amplification for the negative control (B), and 22.71–31.76 for the tested samples (C). The blue horizontal line indicates the fluorescence threshold used to determine Ct values. Internal amplification controls were included in each assay but are not displayed.

ASFV was identified using qPCR based on the region of the *p72* major capsid protein gene *B646L*, as described by King *et al*. [38]. The **International Atomic Energy Agency** (Austria, Vienna) provided DNA ASFV as a positive control, and the no-template control contained nuclease-free water as a negative control. The total volume for all reactions was 20 μL, containing 400 nM of each primer, 250 nM probe, iTaq™ Universal Probes Supermix (Bio-Rad Laboratories, Hercules, CA, USA), which contains iQ Supermix 2×, nuclease-free water, and the DNA sample. DNA amplification was performed at 95°C for 5 minutes as initial denaturation, followed by 40 cycles of denaturation at 95°C for 15 s and annealing at 58°C for 30 s. The qPCR reaction was performed using the CFX96 instrument (Bio-Rad Laboratories, Hercules, CA, USA). Amplification was considered positive at a Cq value of ≤38 and negative at a Cq value of ≥40. Baseline fluorescence was automatically determined by the CFX96™ Real-Time PCR reaction software using default parameters, which define the background fluorescence during non-exponential cycles. Controls, including no-template controls and ASFV-positive controls, were included in every run to ensure assay validity, although these curves are not shown in Figure 1 for clarity.

### PCR amplification of target genes

Five of the 33 ASFV-positive specimens detected by qPCR were selected for sequencing. Selection was based on Ct values, with preference given to samples exhibiting higher Ct values (Ct values of 22.85, 24.78, 23.11, 23.36, and 28.47 from four spleen and one liver samples, respectively). Geographical representation (East Kalimantan, North Sumatra, NTT) was also considered, ensuring the inclusion of strains from different outbreak locations and years. This strategy was adopted to capture broader molecular variation among circulating ASFV strains. PCR was performed on nucleic acids extracted from ASFV-selected PCR-positive samples using specific primers for genetic characterization, which amplified three independent regions of the ASFV genome: 478 bp of the *B646L* (*p72*) gene was amplified using primers p72-U 5′GGCACAAGTTCGGACATGT3′ and p72-D 5′GTACTGTAACGCAGCACAG3′ [28]; the full-length *E183L* gene encoding protein p54 (861 bp) was amplified using primers P54F 5′GCCTGC GGATTCT GAAGATA3′ and P54R 5′AGGACGCAATTGCTTAAACG3′ [39]; and 356 bp of a TRS between the *I73R*/*I329L* genes was amplified using primers ECO1A 5′CCATTTATCCCCCGCTTTGG3′ and ECO1B 5′TCGTCATCCTGAGACAGCAG3′ [40]. In accordance with international ASFV genotyping protocols, only the C-terminal region of the *B646L* (*p72*) gene was sequenced in this study. This partial fragment is the standardized and widely accepted genomic region used to classify ASFV into the 24 recognized genotypes, enabling direct comparison with previously published sequences from Asia, Europe, and Africa. Although full-length p72 sequencing may reveal additional mutations, the partial p72 region provides sufficient resolution for genotype identification and molecular epidemiology and allows consistent comparison with earlier Indonesian and global ASFV datasets. The DNA amplification of each of the three genotypic genes was performed in a 50 µL volume in the presence of 1× PCR buffer 10×, 200 µM dNTPs mix 1 mM MgCl_2_ 500 nM of each primer, 1.25 U Taq polymerase, DNAse-free water, and DNA template. The PCR conditions for amplification of the *B646L* (*p72*) gene included an initial denaturation at 95°C for 5 min, followed by 40 cycles of denaturation at 95°C for 30 s, annealing at 54°C for 30 s, and extension at 72°C for 1 min, and then a final extension at 72°C for 10 min. For the PCR of the *E183L* (*p54*) gene, an initial denaturation was performed at 95°C for 5 min, followed by 15 cycles of denaturation at 95°C for 30 s, annealing at 60°C for 30 s, and extension at 72°C for 1 min, followed by 25 cycles of denaturation at 95°C for 30 s, annealing at 58°C for 30 s, and extension at 72°C for 1 min, and a final extension at 72°C for 5 min. For the IGR between the *I73R*/*I329L* gene, an initial denaturation was performed at 95 °C for 5 min, followed by 40 cycles of denaturation at 95°C for 30 s, annealing at 55°C for 30 s, extension at 72°C for 1 min, and a final extension at 72°C for 7 min. Amplification products were loaded on a 1% agarose gel stained with GelRed® (Biotium, Fremont, CA, USA), along with an Invitrogen 100-bp ladder, and run in an electrophoresis chamber at 100 V/cm for 30 min. The gel was visualized using UVITEC FireReader V10 (Cambridge, England) for the documentation system. All steps were performed as standard for PCR to avoid amplicon contamination.

### Sequencing

The PCR products were sequenced using Sanger sequencing. First, the PCR template is visualized on a gel to confirm the presence of a specific product with the correct size. Confirmation of DNA concentration and purity using a nanodropper. The PCR products were sequenced by Macrogen (Daejeon, South Korea) according to the manufacturer’s instructions. Templates and primers should be prepared according to the manufacturer’s protocol. A representative of Macrogen will pick up our samples, and we will receive an email containing a confidential link to our sequencing results.

### Phylogenetic analysis

The raw sequences were assembled using the default settings in BioEdit. The nucleotide sequences of the Indonesian ASFV strain samples were compared to publicly available sequences using the Basic Local Alignment Search Tool. Multiple alignments of all sequences were performed using the CLUSTAL W algorithm implemented in the BioEdit 7 software package. Sequencing was performed in both forward and reverse directions. Trimming was performed manually based on the shortest sequence in the multiple sequence alignment (MSA), a chromatogram quality with a threshold of 20 (Q20). The ambiguous base was confirmed based on the chromatogram peak and the sequence reference. The assembly of contigs was performed using BioEdit and verified by forward and reverse sequencing. Phylogenetic tree analysis was performed using MEGA software version X to analyze the molecular characteristics of these gene sequences. A neighbor-joining tree of the *B646L* (*p72*) nucleotide sequences was constructed using the Maximum Likelihood method. A minimum evolution (ME) tree of the *E183L* (*p54*) nucleotide sequences was constructed using the Kimura 2-parameter substitution model. The data were resampled 1,000 times using the bootstrap method. Multiple sequence alignments and IGR analysis were performed using BioEdit, as described by Gallardo *et al*. [16]. Phylogenetic tree analysis was performed to analyze the molecular characteristics of these gene sequences based on previous studies by Couacy-Hymann *et al*. [6] and Dharmayanti *et al*. [22]. The sequence was submitted to GenBank in accordance with the GenBank protocol. The gene sequences of the Indonesian ASFV samples from spleen and liver were submitted to the NCBI GenBank database under accession numbers *B646L* (*p72*) (GenBank accession nos. PQ145176, PQ145174, PQ145175, PQ137914, and PQ145173) and *E183L* (*p54*) (GenBank accession nos. PQ145180, PQ145178, PQ145179, PQ145177).

## RESULTS

### ASFV detection in clinical samples

Of the 33 clinical samples evaluated by qPCR using the ASFV primer based on King *et al*. [38], the results showed that the 33 samples from 3 provinces were positive for ASFV based on the *B646L* (*p72*) gene (Figure 1, Table 1). Then, 5 samples were then selected from the 33 positive samples and further confirmed by sequencing using a third-party service (Macrogen, Daejeon, South Korea). The 5 sample codes for sequencing are IDN/2021/Pig/SMADA4, IDN/2022/Pig/PSJ, IDN/2022/Pig/PSB, IDN/2023/Pig/NTT23, and IDN/2023/Pig/NTT32. The gene sequences of the Indonesian ASFV samples from spleen and liver were submitted to the NCBI GenBank database under accession numbers *B646L* (*p72*) (GenBank accession nos. PQ145176, PQ145174, PQ145175, PQ137914, and PQ145173) and *E183L* (*p54*) (GenBank accession nos. PQ145180, PQ145178, PQ145179, PQ145177) (Table 2).

**Table 2:** GenBank accession numbers of ASFV strains, Indonesia, 2021–2023.

Sample code	Year	Strain	Sample type	TRS	*E183L* (*p54*)	GenBank accession number *B646L* (*p72*)	GenBank accession number *E183L* (*p54*)
SMADA4	2021	IDN/2021/Pig/SMADA4	Spleen	GGAATATATA	–	PQ145176	PQ145180
PSJ	2022	IDN/2022/Pig/PSJ	Spleen	GGAATATATA	PVTDN	PQ145175	PQ145178
PSB	2022	IDN/2022/Pig/PSB	Spleen	GGAATATATA	PVTDN	PQ145174	PQ145179
NTT23	2023	IDN/2023/Pig/NTT23	Spleen	GGAATATATA	–	PQ137914	–
NTT32	2023	IDN/2023/Pig/NTT32	Liver	GGAATATATA	–	PQ145173	PQ145177

### Phylogenetic analysis of the *B646L* (*p72*) gene

**Figure 2:**
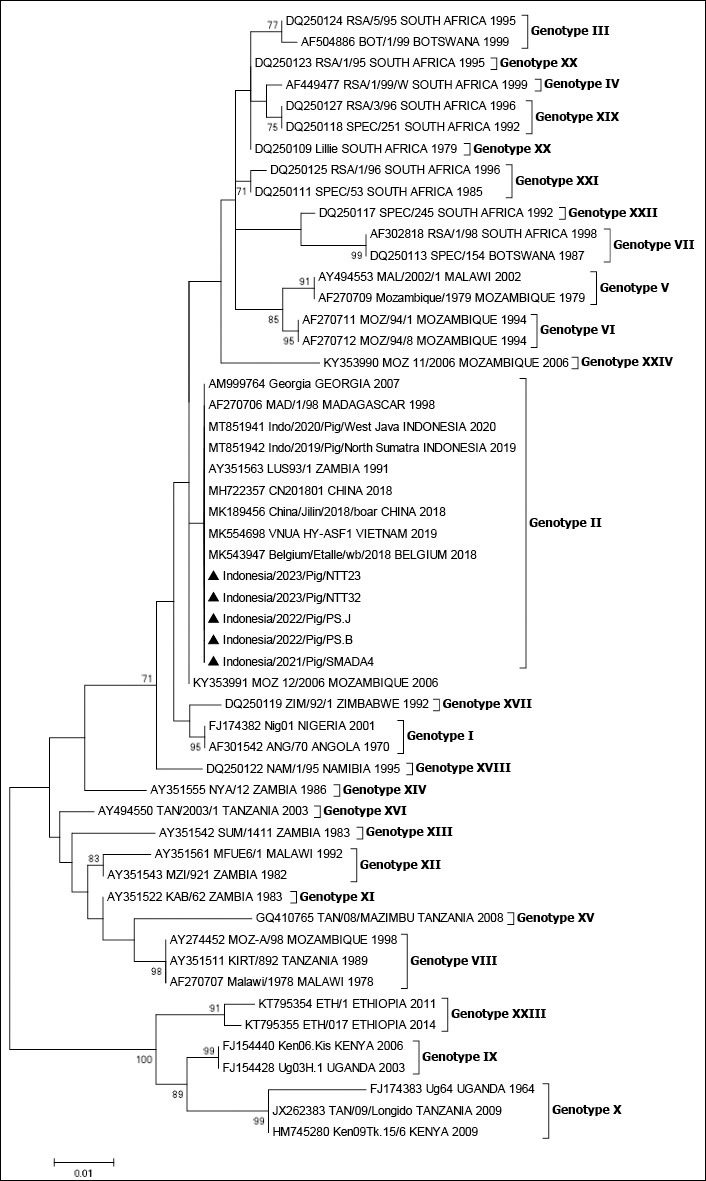
Phylogenetic analysis of ASFV isolates based on the *B646L* (*p72*) gene. Phylogenetic tree showing the relationship of ASFV isolates identified in this study (marked with a black triangle, ▲) within the global ASFV genotype constellation. The tree was constructed using the Neighbor-Joining method with 1,000 bootstrap replicates. Only bootstrap values greater than 70% are shown. Nucleotide sequences were aligned using BioEdit version 7, and GenBank accession numbers are provided for all sequences. The scale bar represents nucleotide substitutions per site.

We amplified, sequenced, and analyzed the *B646L* (*p72*) gene. Phylogenetic analysis of the partial C-terminus region of the *B646L* (*p72*) nucleotide sequences revealed that all ASFVs, including the five samples obtained in this study and the reference strains downloaded from GenBank, could be clustered into 24 genotypes. The five samples from this study were genotype II. Phylogenetic analysis of the partial C-terminus region of the *B646L* (*p72*) gene sequences confirmed the circulation of genotype II (Figure 2). The results of amino acid analysis of the *B646L* (*p72*) gene revealed no amino acid variation between the isolates used in this study and other Indonesian isolates (Table 3).

**Table 3:** Amino acid sequences of the *B646L* (*p72*) gene among Indonesian ASFV isolates compared with other Indonesian isolates.

Year	Accession number	Strain code	Sample origin	1	2	3	4	5	6	7	8	9	10	…	125	126	127	128	129	130	131	132	133	134
2019	MT851942	IDN/2019/Pig/North Sumatra	North Sumatra	M	Q	P	T	H	H	A	E	I	S	…	D	L	V	V	S	A	S	A	I	N
2019	OR428152	IDN/Pig/North Sumatera/A01190497	North Sumatra	M	Q	P	T	H	H	A	E	I	S	…	D	L	V	V	S	A	S	A	I	N
2019	OR428151	IDN/pig/North Sumatera/A01190069	North Sumatra	M	Q	P	T	H	H	A	E	I	S	…	D	L	V	V	S	A	S	A	I	N
2019	PP869833	IDN/2019/T05	Jakarta	M	Q	P	T	H	H	A	E	I	S	…	D	L	V	V	S	A	S	A	I	N
2019	PP869832	IDN/2019/T04	Jakarta	M	Q	P	T	H	H	A	E	I	S	…	D	L	V	V	S	A	S	A	I	N
2020	MT851941	IDN/2020/Pig/West Java	West Java	M	Q	P	T	H	H	A	E	I	S	…	D	L	V	V	S	A	S	A	I	N
2020	OR428154	IDN/Pig/North Sumatera/P01200055	North Sumatra	M	Q	P	T	H	H	A	E	I	S	…	D	L	V	V	S	A	S	A	I	N
2020	OR428153	IDN/Pig/North Sumatera/A01200500	North Sumatra	M	Q	P	T	H	H	A	E	I	S	…	D	L	V	V	S	A	S	A	I	N
2021	OR428156	IDN/Pig/North Sumatera/A01211590	North Sumatra	M	Q	P	T	H	H	A	E	I	S	…	D	L	V	V	S	A	S	A	I	N
2021	OR428155	IDN/Pig/North Sumatra/A012111523	North Sumatra	M	Q	P	T	H	H	A	E	I	S	…	D	L	V	V	S	A	S	A	I	N
2022	OR428158	IDN/Pig/North Sumatera/A01222480	North Sumatra	M	Q	P	T	H	H	A	E	I	S	…	D	L	V	V	S	A	S	A	I	N
2022	OR428157	IDN/Pig/North Sumatera/A01222297	North Sumatra	M	Q	P	T	H	H	A	E	I	S	…	D	L	V	V	S	A	S	A	I	N
2023	OR428159	IDN/Pig/North Sumatera/AR127501230112	North Sumatra	M	Q	P	T	H	H	A	E	I	S	…	D	L	V	V	S	A	S	A	I	N
2021	PQ145176	IDN/2021/Pig/SMADA4	East Kalimantan	M	Q	P	T	H	H	A	E	I	S	…	D	L	V	V	S	A	S	A	I	N
2022	PQ145174	IDN/2022/Pig/PS.J	North Sumatra	M	Q	P	T	H	H	A	E	I	S	…	D	L	V	V	S	A	S	A	I	N
2022	PQ145175	IDN/2022/Pig/PS.B	North Sumatra	M	Q	P	T	H	H	A	E	I	S	…	D	L	V	V	S	A	S	A	I	N
2023	PQ137914	IDN/2023/Pig/NTT23	NTT	M	Q	P	T	H	H	A	E	I	S	…	D	L	V	V	S	A	S	A	I	N
2023	PQ145173	IDN/2023/Pig/NTT32	NTT	M	Q	P	T	H	H	A	E	I	S	…	D	L	V	V	S	A	S	A	I	N

*Amino acids are abbreviated using IUPAC standard codes. Numbers in the header row indicate amino acid positions in the *B646L* (*p72*) protein sequence.

### Phylogenetic analysis of *E183L* (*p54*)

**Figure 3:**
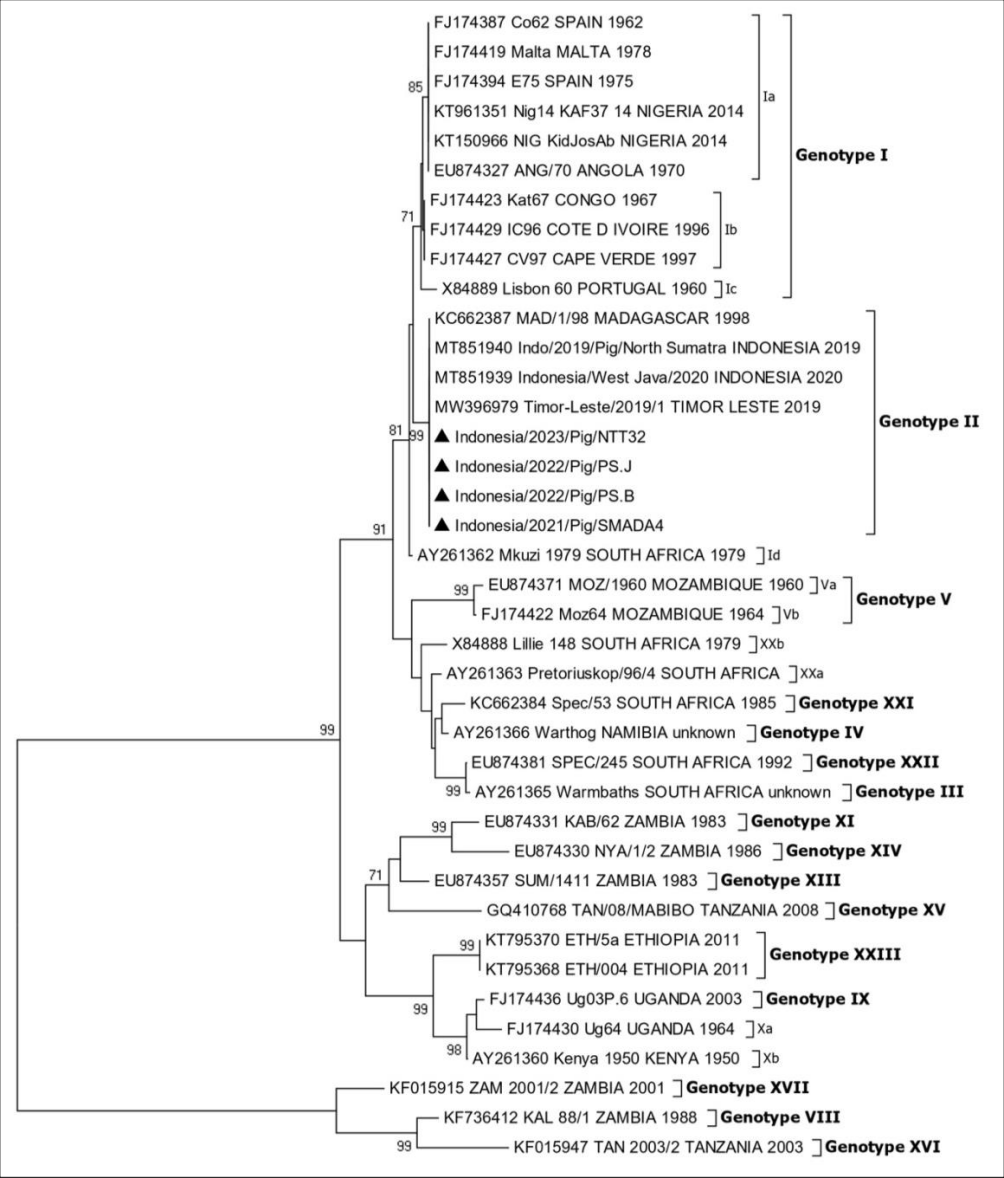
Phylogenetic analysis of ASFV isolates based on the *E183L* (*p54*) gene. Phylogenetic tree illustrating the genetic relationships of ASFV isolates from this study (▲) within the ASFV genotype constellation. The tree was generated using the minimum evolution method with the Kimura two-parameter model in MEGA X and 1,000 bootstrap replicates. Only bootstrap values greater than 70% are displayed. Nucleotide sequence alignment was performed using BioEdit version 7. GenBank accession numbers are provided for all sequences. The scale bar indicates nucleotide substitutions per site.

We amplified, sequenced, and analyzed the *E183L* (*p54*) gene. Because all five samples from the three provinces belonged to genotype II based on the phylogenetic analysis of the *B646L* (*p72*) gene, we determined whether these strains also belonged to genotype II based on the phylogenetic analysis of the *E183L* (*p54*) gene. The results revealed that all five samples from this study and the reference strains downloaded from GenBank could be clustered into different genotypes and sub-genotypes. All five samples in this study were genotype II (Figure 3). Full-length *E183L* (*p54*) nucleotide sequence analysis confirmed that the virus responsible for the 2021-2023 outbreak belongs to genotype II (Figure 3). The results of amino acid analysis of the *E183L* (*p54*) gene showed that two Indonesian samples, IDN/2020/Pig/PSJ and IDN/2020/Pig/PSB, had genetic variations in the form of insertions of five amino acids (PVTDN) compared with other Indonesian strains in GenBank (Table 4). The results of amino acid analysis of the *E183L* (*p54*) gene in genotype II showed that only the Indonesian samples IDN/2020/Pig/PSJ and IDN/2020/Pig/PSB had genetic variations in the form of insertions of five amino acids with the PVTDN motif compared with the strains from Indonesia and other countries (Table 5). In Tables 4 and 5, numerical labels (e.g., 1, 2, 3, 125) refer to the corresponding amino acid positions in the *E183L* (*p54*) protein used to compare residue variations and identify the PVTDN insertion.

**Table 4:** Amino acid sequences of the *E183L* (*p54*) gene among Indonesian samples analyzed in this study compared with other Indonesian strains.

Accession number	Strain code	Sample origin	115	116	117	118	119	120	121	122	123	124	125	126	127	128	129	129	130
OR539714	IDN/069/2019	North Sumatra	K	P	V	T	D	N	-	-	-	-	-	P	V	T	D	R	L
OR539715	IDN/0497/2019	North Sumatra	K	P	V	T	D	N	-	-	-	-	-	P	V	T	D	R	L
MT851940	IDN/2019/Pig/North Sumatra	North Sumatra	K	P	V	T	D	N	-	-	-	-	-	P	V	T	D	R	L
PP869834	IDN/2019/T04	Jakarta	K	P	V	T	D	N	-	-	-	-	-	P	V	T	D	R	L
MT851939	IDN/2020/Pig/West Java	West Java	K	P	V	T	D	N	-	-	-	-	-	P	V	T	D	R	L
OQ061169	Pig/IDN/Lampung Timur/2020	Lampung	K	P	V	T	D	N	-	-	-	-	-	P	V	T	D	R	L
OQ061161	Boar/IDN/Muara Enim/2021	South Sumatra	K	P	V	T	D	N	-	-	-	-	-	P	V	T	D	R	L
OQ061163	Pig/IDN/Lampung Selatan/2020	Lampung	K	P	V	T	D	N	-	-	-	-	-	P	V	T	D	R	L
OQ061164	Pig/IDN/Lampung Selatan/2020	Lampung	K	P	V	T	D	N	-	-	-	-	-	P	V	T	D	R	L
OR539718	IDN/1523/2021	North Sumatra	K	P	V	T	D	N	-	-	-	-	-	P	V	T	D	R	L
OR539719	IDN/1590/2021	North Sumatra	K	P	V	T	D	N	-	-	-	-	-	P	V	T	D	R	L
OQ061168	Pig/IDN/Lampung Tengah/2022	Lampung	K	P	V	T	D	N	-	-	-	-	-	P	V	T	D	R	L
OR539720	IDN/2297/2022	North Sumatra	K	P	V	T	D	N	-	-	-	-	-	P	V	T	D	R	L
OR539722	IDN/2480/2022	North Sumatra	K	P	V	T	D	N	-	-	-	-	-	P	V	T	D	R	L
OR539721	IDN/112/2023	North Sumatra	K	P	V	T	D	N	-	-	-	-	-	P	V	T	D	R	L
PQ145180	IDN/2021/Pig/SMADA4	East Kalimantan	K	P	V	T	D	N	-	-	-	-	-	P	V	T	D	R	L
PQ145178	IDN/2022/Pig/PS.J	North Sumatra	K	P	V	T	D	N	P	V	T	D	N	P	V	T	D	R	L
PQ145179	IDN/2022/Pig/PS.B	North Sumatra	K	P	V	T	D	N	P	V	T	D	N	P	V	T	D	R	L
PQ145177	IDN/2023/Pig/NTT32	NTT	K	P	V	T	D	N	-	-	-	-	-	P	V	T	D	R	L

*Amino acids are abbreviated using IUPAC standard codes. Numbers in the header row represent amino acid positions in the E183L (p54) protein sequence.

**Table 5:** The amino acid sequences of the *E183L* (*p54*) gene between Indonesian samples analyzed in this study were compared with those of strains from Indonesia and other countries.

Genotype	Strain code	Nation	Amino acid sequence (positions 107–143)
Ia	Co62	ESP	P A T N R – – – – – – P A T N K P V T D N – – – – – – – – – – P V T D R L
Ib	Kat67	CGO	P A T N R – – – – – – P A T N K P V T D N – – – – – – – – – – P V T D R L
Ic	Lisbon 60	PRT	P A T N R – – – – – – P A T N K P V T D N – – – – – – – – – – P V T D R L
Id	Mkuzi 1979	SA	P A T N R – – – – – – P A T N K P V T D N – – – – – – – – – – P V T D R L
II	Georgia 2007	GEO	P A T N R – – – – – – P A T N K P V T D N – – – – – – – – – – P V T D R L
II	CN201801	CHN	P A T N R – – – – – – P A T N K P V T D N – – – – – – – – – – P V T D R L
II	VNUA HY-ASF2	VNM	P A T N R – – – – – – P A T N K P V T D N – – – – – – – – – – P V T D R L
II	IDN/2022/Pig/PS.J	IDN	P A T N R – – – – – – P A T N K P V T D N **P V T D N** – – – – – P V T D R L
II	IDN/2023/Pig/NTT32	IDN	P A T N R – – – – – – P A T N K P V T D N – – – – – – – – – – P V T D R L
III	Warmbaths	SA	P A T N R – – – – – – L V A D R P A T N R – – – – – – – – – – P V M D M P
IV	Warthog	NA	P A T N R – – – – – – P V T D R P A T N N – – – – – – – – – – P V T D R L
Va	MOZ/1960	MOZ	P A T N R – – – – – – – – – – – – – – – – – – – – – – – – – – P V T D R L
Vb	Moz64	MOZ	P A T N R – – – – – – – – – – – – – – – – – – – – – – – – – – P V T D R L
VIII	KAL 88/1	ZM	P V T N K – – – – – – P V T N K P V T D R – – – – – – – – – – – – – – – L
IX	Ug03P.6	UGA	P A T D R – – – – – – P V T N S S A V D R – – – – – P V M N S P V T D R L
Xa	Ug64	UGA	P A T D R P V A M N R P V T N S S A V D R P V M N N P V T D S P V T D R L
Xb	Kenya 1950	KE	P A T N R – – – – – – – – – – – – – – – – – – – – – – – P V T N N P V T D R L
XI	KAB/62	ZM	P V T N K – – – – – – P I T N K P V T N N – – – – – – – – – – P V T D R L
XIII	SUM/1411	ZM	P V T N K – – – – – – P V T N K P V T N N P V T N N P V I N N S V T D R L
XIV	NYA/1/2	ZM	P V T N R – – – – – – P A T N N P I T D R – – – – – – – – – – – – – – – L
XV	TAN/08/MABIBO	TZA	P I T N N – – – – – – P V M D K P V T N H P V T D R – – – – – L V T D K L
XVI	TAN 2003/2	TZA	P V T N N – – – – – – P V T N N P V T N N P V T N N – – – – – P V T D R L
XVII	ZAM 2001/2	ZM	P A T N R – – – – – – P A T N K P V T D N – – – – – – – – – – P V T D R L
XXa	Pretoriuskop/96/4	SA	P A T N R – – – – – – P V T D R P A T N N – – – – – – – – – – P V T D R L
XXb	Lillie 148	SA	P A T K R – – – – – – P A T D R P A T N N – – – – – – – – – – P V T D R L
XXI	Spec/53	SA	P A T N R – – – – – – P V T D R P A T N N – – – – – – – – – – P V T D R L
XXII	SPEC/245	SA	P A T N R – – – – – – L V A D R P A T N R – – – – – – – – – – P V M D N P
XXIII	ETH/5a	ET	P A T D R – – – – – – P V T N S P V T N R – – – – – L V T N S P V T D R L

*Amino acids are abbreviated using IUPAC standard codes. Numbers in the header row represent amino acid positions in the E183L (p54) protein sequence.

### Analysis of IGR between the *I73R*/*I329L* gene

**Figure 4:**
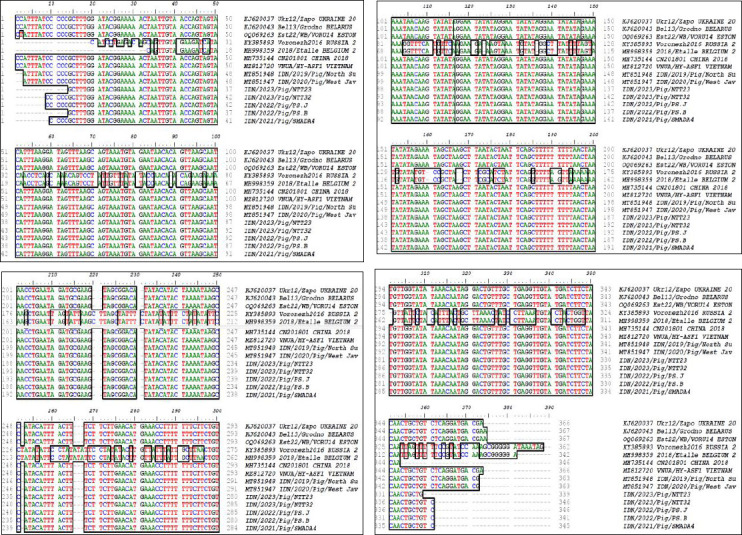
Alignment of the intergenic region between *I73R* and *I329L* genes of ASFV isolates.Multiple-sequence alignment of the intergenic region between the *I73R* and *I329L* genes among Indonesian ASFV isolates analyzed in this study and genotype II reference strains from Indonesia and other countries. The red box indicates the tandem repeat sequence insertion motif GGAATATATA, and black boxes represent individual tandem repeat sequence units located in the intergenic region. The presence of two tandem repeat sequence units corresponds to the IGR II variant characteristic of ASFV genotype II. The insertion pattern observed in this study is identical to those reported in ASFV isolates from domestic pigs in China (MH735144), Vietnam (MZ812720), Belarus (KJ620043), Estonia (OQ069263), and Ukraine (KJ620037), but differs from strains reported in Russia (KY385893) and Belgium (MH998359).

The IGR between the *I73R*/*I329L* genes was amplified, sequenced, and characterized by the presence of a TRS. TRS variants were identified by analyzing the IGR between the *I73R*/*I329L* genes of the ASFV genome. Classification into IGR I-IGR IV was based on the presence and number of 10-bp tandem repeat insertions relative to a reference strain. Sequence comparisons were performed using the ASFV China CN201801 2007 strain (MH735144) as the reference strain in Asia, representing the IGR II configuration. The raw nucleotide sequences were assembled and trimmed before analysis. Automated multiple sequence alignment was performed using a standard alignment algorithm (ClustalW) implemented in BioEdit v.7 sequence analysis software. The aligned sequences were then screened for insertions or deletions within the *I73R*/*I329L* IGR, with a focus on the characteristic 10-bp TRS motif. Automated alignment against the reference strain was used to identify TRS variants. The TRS with the GGAATATATA motif within the IGR between *I73R* and *I329L* (*I73R*/*I329L*) in genotype II was 100% identical between the Indonesian samples analyzed in this study and the Indonesian strain from the early 2019 outbreak. All five samples in this study belonged to IGR II, characterized by two 10-nucleotide GGAATATATA insertions (Figure 4). Each black box in Figure 4 denotes one GGAATATATA TRS unit in the IGR between the *I73R*/*I329L* genes. All Indonesian isolates contained two identical TRS units, consistent with the IGR II variant described for genotype II ASFV. The TRS insertion with the GGAATATATA motif was also identical to that of ASFV strains from domestic pigs in Vietnam (MZ812720), Belarus (KJ620043), Estonia (OQ069263), and Ukraine (KJ620037); however, it was not found in virus strains from Russia (KY385893) and Belgium (MH998359).

## DISCUSSION

### ASF epidemiology and spread in Indonesia

ASF remains a significant challenge in pig production in Indonesia, with sporadic outbreaks occurring throughout the year. Since its initial outbreak in North Sumatra Province in 2019 [22], the disease has become endemic. The introduction of ASF to Indonesia is suspected to have occurred via contaminated products from infected countries through international transportation pathways, such as international air flights [35]. According to the FAO, ASF has been officially reported in 32 of the 38 provinces in Indonesia, highlighting its widespread impact [3]. To better understand disease outbreak patterns and map viral genotypes to specific geographical regions, ongoing characterization of ASFV samples during outbreaks is critical. This continuous genotyping process is essential for gaining insights into the origin of the virus during each outbreak, thereby enhancing our epidemiological understanding of the disease and potentially informing more effective control strategies.

### Molecular approaches for ASFV characterization

The molecular characterization of distinct ASFV genome regions has contributed to the understanding of the origins and transmission pathways of ASF during outbreak scenarios. The prevailing approach in studying the molecular epidemiology of ASF involves sequence analysis of the 3’-end of the *B646L* gene, which encodes the p72 protein, and at least 24 ASFV genotypes. Comprehensive sequence analysis of the *E183L* gene, which encodes the envelope protein p54, provides an intermediate level of viral discrimination, facilitating the classification of isolates into major subgroups. The phylogenetic analysis of *E183L* (*p54*) generally aligns with the pattern of *B646L* (*p72*). Additionally, examining other genomic regions situated between the *I73R*/*I329L* genes may enhance the discrimination of closely related ASFV strains [26].

### Genotype confirmation in Indonesian outbreaks

The first characterized ASFV in Indonesia was genotype II based on sequencing of the C-terminal region of the *B646L* (*p72*) gene [22]. Further differentiation between close strains and identification of virus subtypes for the 24 genotypes were achieved by nucleotide sequencing of the whole *E183L* gene, specifically the regions encoding p54 proteins. Despite the presence of many other marker genes, previous studies have shown that using the three encoding regions of ASFV DNA, including *B646L* (*p72*), *E183L* (*p54*), and IGR between *I73R*/*I329L* genes, to characterize ASFV is sufficient [13, 29, 32].

### Outbreak history in the studied provinces

According to reports from the provincial veterinary service, the initial occurrence of ASF on a backyard pig farm in Berau City, East Kalimantan Province, was reported in July 2021. In November 2022, a subsequent outbreak was reported in Pematang Siantar city, North Sumatra Province, also on a backyard pig farm. In March 2023, the third outbreak was reported in Kupang city, Nusa Tenggara Timur (NTT), affecting a backyard pig farm for the second time. The disease affects the domestic pig population and is characterized by symptoms including fever (42 °C), dyspnea, nasal discharge, anal bleeding, and anorexia.

### Detection and sample characteristics

A total of 33 samples were obtained from three provinces that tested positive for ASFV through qPCR analysis targeting the *B646L* (*p72*) gene (Figure 1 and Table 2). These samples exhibited a range of Ct values, ranging from 22.71 to 31.76, indicating varying viral loads. Lower Ct values were correlated with high viral load and a specific sample type (i.e., spleen). ASFV poses a significant threat to backyard pig farms due to inadequate biosecurity measures. Pigs are typically housed in open or semi-enclosed areas in these farms, increasing the likelihood of direct interaction with wild pigs or exposure to contaminated areas. The use of shared equipment, inadequate disinfection, feeding pigs with swill, and frequent movement of people and animals on and off the farm further intensify the potential for ASFV transmission. In addition, backyard farmers may lack the knowledge or resources to promptly detect and manage an outbreak, allowing the virus to spread undetected [41, 42].

### Genotype II confirmation and p72 conservation

The phylogenetic analysis of ASFV samples collected during outbreaks in Indonesia from 2021 to 2023 provides valuable insights into the genetic characteristics of circulating strains. We determined the genotype of the viruses by examining the *B646L* (*p72*) gene sequences from positive clinical samples obtained from East Kalimantan, North Sumatra, and NTT Provinces. The analysis revealed that all five samples from these three provinces belonged to genotype II (Figure 2), consistent with the findings from the 2019 outbreak in North Sumatra Province and other countries in Asia [22, 27, 34]. The results of amino acid analysis of the *B646L* (*p72*) gene showed no amino acid variation between the samples used in this study and other Indonesian strains (Table 3). Amino acid analysis of the *B646L* (*p72*) gene revealed complete conservation among the samples examined in this study and other Indonesian strains. This finding suggests a high degree of genetic stability in the p72 protein sequence across different ASFV strains circulating in Indonesia. The absence of amino acid variations indicates that this particular gene region may be under strong selective pressure to maintain its functional integrity, possibly because of its critical role in viral replication or structural stability. This conservation of the *B646L* (*p72*) gene sequence has important implications for ASFV diagnostic and epidemiological studies in Indonesia and other countries. From a diagnostic perspective, the consistency in amino acid composition supports the continued use of *B646L* (*p72*)-based detection methods, as they are likely to remain effective across various Indonesian ASFV samples.

### Enhanced resolution with *E183L* (*p54*) and novel insertion

Complete sequence analysis of the *E183L* (*p54*) gene from four ASFV samples collected across three Indonesian provinces between 2021 and 2023 classified them as genotype II (Figure 3). This aligns with earlier research indicating that the ASFV responsible for the initial outbreak in Indonesia, as well as in various parts of Southeast Asia, belongs to genotype II [22]. The *E183L* (*p54*) gene enhances the intragenotypic resolution of the standard p72 genotype, thereby improving genotype identification accuracy. The phylogenetic trees derived from the *B646L* (*p72*) and *E183L* (*p54*) genes were identical, and the *E183L* (*p54*) genotype offered further subdivision within the largest branch of p72 genotype I, divided into four subclusters: Ia, Ib, Ic, and Id [27, 29]. The *E183L* (*p54*) gene in ASFV shows genetic variations among different samples, particularly in Indonesian samples (Table 4). The IDN/2020/Pig/PSJ and IDN/2020/Pig/PSB samples from Indonesia had a unique insertion of five amino acids with the PVTDN motif, distinguishing them from other Indonesian and global strains. This genetic variation highlights the potential for regional adaptations within the virus and the importance of continuous surveillance to monitor strain evolution [24]. Interestingly, similar 5-amino acid insertions with slightly different motifs have been identified in strains from Tanzania, Uganda, and Zambia (Table 5). Insertions of 5 amino acids are found in the Tanzanian strain TAN/08/MABIBO (genotype XV), featuring a PVTDR motif, and strain TAN2003/2 (genotype XVI), with the PVTNN motif. Insertions of 5 amino acids are also found in the Ugandan strain Ug64 (genotype Xa), with the PVMNN motif, and the Zambian strain SUM/1411 (genotype XIII), with the PVTNN motif. A PVTDN was identified at positions 121–125 of the *E183L* (*p54*) protein in two isolates from North Sumatra. Sequence comparison revealed that this insertion is a tandem duplication of the adjacent KPVTDN motif, resulting in the expanded KPVTDN–PVTDN–PVTDR motif. This region lies within a proline-/alanine-rich segment of p54 that may participate in intracellular trafficking or protein–protein interactions. However, the specific functional consequences of such duplications in genotype II strains remain unknown. Because our study did not include phenotypic characterization, the evolutionary or biological significance of this micro-variation cannot be inferred and warrants further investigation. A notable finding of this study is the identification of a five amino acid insertion (PVTDN) in the *E183L* (*p54*) gene in two isolates from North Sumatra. This represents the first documentation of such an insertion in Indonesian ASFV genotype II strains. Comparative analysis showed that the insertion corresponds to a tandem duplication of the adjacent KPVTDN motif, located at amino acid positions 121–125, producing the expanded motif KPVTDN–PVTDN–PVTDR. This insertion differentiates the North Sumatra isolates from all other Indonesian strains reported between 2019 and 2023, as well as from all publicly available Southeast Asian genotype II sequences, which uniformly lack this duplication. Similar-sized insertions have been described in African genotypes XV, XVI, and XIII; however, these African insertions appear identical to the Indonesian duplication alignment, demonstrating that the Indonesian PVTDN duplication is distinct from African insertions and almost certainly arose independently. This suggests a localized microevolutionary event within the outbreak cluster in North Sumatra. The biological implications of this duplication remain unclear. The affected region lies within a proline- and alanine-rich segment of p54 thought to participate in intracellular transport and host-protein interactions. While insertions in this region could theoretically influence protein conformation, stability, or host interaction, no experimental evidence is available to determine functional consequences. The PVTDN duplication may represent a neutral evolutionary event rather than an adaptive change given the limited number of isolates displaying this feature. Further functional characterization will be required to evaluate its virological significance. Overall, the discovery of the PVTDN insertion constitutes a novel molecular signature within Indonesian ASFV, providing new insights into possible microevolution among genotype II isolates circulating in North Sumatra. The PVTDN motif insertions in the ASFV *E183L* gene represent genetic variability within the viral genome. These insertions, which occur independently across different geographical regions and genotypes, highlight the dynamic nature of ASFV evolution. Variations in diverse isolates enhance the virus’s adaptability, potentially enabling it to thrive in various host environments or respond to different selective pressures. This gene encodes a structural protein involved in virus assembly, and variations in its sequence could affect viral replication efficiency, virulence, or host immune recognition. Understanding these variations could provide valuable insights into the biology of ASFV and inform strategies for disease control and vaccine development [43, 44]. The presence of an insertion of 5 amino acids with the PVTDN motif in 2 ASFV sample strains (GenBank accession numbers PQ145178, PQ145179) originating from North Sumatra, based on the *E183L* (*p54*) gene, is important information for further research for the development of vaccine candidates based on local isolates, to increase vaccine effectiveness.

### IGR analysis and genetic stability

The intergenic region (IGR) sequence between the *I73R*/*I329L* genes was analyzed to determine the relationship between relevant genotypes and the origin of circulating ASFV strains [13]. The IGR between the *I73R*/*I329L* genes was amplified, sequenced, and characterized because of the presence of a TRS. This study revealed genetic similarities among ASFV strains in Indonesia, particularly in the TRS, which features the GGAATATATA motif. This TRS, located in the IGR between the *I73R*/*I329L* genes, was 100% identical across all Indonesian samples. The analysis of the sequences obtained between the *I73R*/*I329L* genes also exhibited genetic stability [45]. The results of the analysis performed between the *I73R*/*I329L* genes are consistent with those reported in a previous study in which we analyzed two strains collected between 2019 (IDN/2019/Pig/North Sumatra) and 2020 (IDN/2020/Pig/West Java). These samples, classified as IGR II, each contained two 10-nucleotide GGAATATATA insertions. This genetic consistency suggests a common lineage or recent introduction of the virus in the region, potentially indicating a single source of infection or limited genetic diversity among circulating strains. To the best of our knowledge, this is the first study to document the multi-year genetic stability of ASFV genotype II in Indonesia, demonstrating the persistence of an identical *B646L* (*p72*) sequence and a conserved IGR II TRS motif across outbreaks spanning 2021–2023. Interestingly, this study also highlighted the genetic relationships between ASFV strains from Indonesia and those from other countries. The TRS insertion with the GGAATATATA motif observed in Indonesian samples was identical to that in ASFV genotype II strains circulating in Vietnam [46], China [13, 20], Belarus, Estonia, and Ukraine [13, 17]. However, this specific genetic feature was absent in the Russian and Belgian virus strains. This pattern of genetic similarity and differences across geographical regions provides valuable insights into the potential routes of virus transmission and evolution. This may also have implications for understanding the epidemiology of ASFV and developing targeted control strategies based on the genetic characteristics of circulating strains [47].

### Implications for backyard farming and control

ASF poses a significant threat to the pig industry, particularly in developing countries, such as Indonesia. Similar to other Asian countries, small-scale backyard farming with minimal or no biosecurity is the predominant practice in Indonesia, making it the most vulnerable to disease. The practical recommendations for backyard systems include restricting animal movement, improving hygiene practices, avoiding swill feeding, enhancing farmer awareness, and implementing basic quarantine measures for newly introduced animals. Pigs are a significant source of income, especially for smallholder communities, and with an increasing human population, they can potentially help mitigate the risks of food insecurity. Relevant stakeholders should be educated about the disease and the implementation of biosecurity measures to mitigate risks [3, 48, 49]. Continuous genomic surveillance is crucial for identifying emerging strains and understanding their epidemiological impact, as demonstrated by the rapid spread of ASF in Indonesia since its first outbreak in 2019.

## CONCLUSION

This study provides the first multi-province and multi-year molecular characterization of ASFV isolates responsible for outbreaks in East Kalimantan, North Sumatra, and East Nusa Tenggara provinces of Indonesia between 2021 and 2023. All 33 clinical specimens tested positive for ASFV by qPCR targeting the *B646L* (*p72*) gene. Phylogenetic analyses of the partial *B646L* (*p72*) and complete *E183L* (*p54*) genes confirmed exclusive circulation of genotype II. A previously unreported five amino acid insertion (PVTDN motif) was identified at positions 121–125 in the *E183L* (*p54*) protein of the two North Sumatran isolates, representing a novel molecular marker unique to Indonesian genotype II strains. Sequence analysis of the intergenic region between *I73R* and *I329L* (*I73R*/*I329L*) revealed 100% identity of the IGR II variant (two GGAATATATA tandem repeat units) across all samples and with the initial 2019 North Sumatran outbreak, demonstrating sustained spatial and temporal genetic stability of ASFV in Indonesia.

The identification of the PVTDN insertion in *E183L* (*p54*) offers a valuable epidemiological marker for lineage tracing and enhanced surveillance in North Sumatra. The observed genetic homogeneity supports the hypothesis of a single successful introduction followed by limited microevolution, which has direct relevance for the design of regionally tailored diagnostics, vaccines, and control programs. These data strengthen risk assessments for backyard pig systems and inform targeted biosecurity interventions to mitigate further spread across Indonesian islands and neighboring Southeast Asian countries.

The major strength lies in the integrated use of three established genotyping markers (*B646L* (*p72*), *E183L* (*p54*), and *I73R*/*I329L* IGR) applied to samples from three geographically distant provinces over a three-year period. This multi-locus, multi-province, multi-year approach provides higher resolution than previous single-outbreak or single-gene studies conducted in Indonesia and represents the most comprehensive molecular dataset for ASFV genotype II in the country to date.

The study is constrained by the relatively small number of sequenced isolates, the retrospective nature of sample collection from remote backyard farms, and the absence of whole-genome sequencing or paired epidemiological tracing data. Phenotypic characterization of the novel PVTDN insertion was not performed, precluding assessment of its potential biological impact.

Future studies should prioritize whole-genome sequencing of additional ASFV isolates from a wider geographic and temporal range, functional evaluation of the PVTDN insertion in *E183L* (*p54*), and longitudinal surveillance to monitor potential emergence of new variants. Integration of genomic data with detailed epidemiological tracing will further refine transmission models and support evidence-based ASF control strategies in archipelagic settings.

The findings confirm the continued dominance of a genetically stable genotype II ASFV population in Indonesia while documenting a unique molecular signature that may aid future outbreak investigations. This work establishes an essential baseline for ongoing ASF surveillance and highlights the critical need for sustained molecular monitoring to safeguard pig production and food security in the region.

## DATA AVAILABILITY

The supplementary data can be made available from the corresponding author upon request.

## AUTHORS’ CONTRIBUTIONS

AR, RH, and IS: Supervised field sampling, data collection, and laboratory work. AR, RH, IS, SS, MS, ST, HN, and NLPIM: Data entry, analysis, interpretation, and drafting of the manuscript. AR, NLPIM, NLPID, and IWTW: Conceptualized and designed the study, reviewed, and edited the manuscript. All authors have read and approved the final version of this manuscript.
